# Development and Validation of a Test Mold for Thermoplastic Resin Transfer Molding of Reactive PA-6

**DOI:** 10.3390/polym12040976

**Published:** 2020-04-22

**Authors:** Róbert Boros, Ilya Sibikin, Tatyana Ageyeva, József Gábor Kovács

**Affiliations:** Department of Polymer Engineering, Faculty of Mechanical Engineering, Budapest University of Technology and Economics, Műegyetem rkp. 3, 1111 Budapest, Hungary; borosr@pt.bme.hu (R.B.); sibikini@pt.bme.hu (I.S.); ageyevat@pt.bme.hu (T.A.)

**Keywords:** thermoplastic resin transfer molding, mold design, reactive polyamide, thermoplastic composite

## Abstract

Thermoplastic resin transfer molding (T-RTM) is a cutting-edge manufacturing technique for high-volume production of composites with a recyclable thermoplastic matrix. Although a number of reactive thermoplastic matrices as well as industrial manufacturing equipment for T-RTM are commercially available today, the design of a T-RTM mold is still based on the skills and personal experience of the designer. This study summarizes the best knowledge and expertise in mold design and manufacturing and introduces an innovative mold for T-RTM. A concept and basic principles for designing a T-RTM mold are formulated in this study. The mold developed is manufactured and validated.

## 1. Introduction

Modern automobiles must comply with the rigorous environmental regulations and it is not surprising that weight reduction and recyclability are of paramount importance for automakers. At the same time, car structures must withstand significant static and dynamic loads under a great variety of environmental conditions. Fiber-reinforced composites with polymer matrices are materials that can meet these requirements.

**Figure 1:**
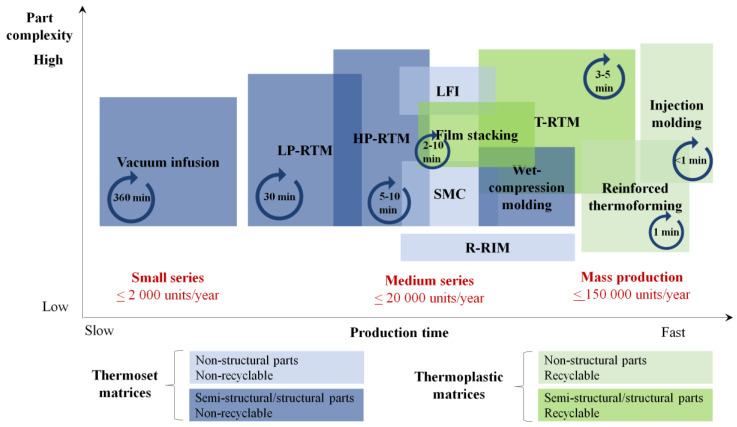
Comparison of various polymer composite production techniques (LP-RTM—Low Pressure Resin Transfer Molding; HP-RTM—High Pressure Resin Transfer Molding; LFI—Long Fiber Injection; SMC—Sheet Molding Compound; R-RIM—Reinforced Resin Injection Molding; T-RTM—Thermoplastic Resin Transfer Molding).

Basically, there are two kinds of polymer matrices: thermoset and thermoplastic. Until very recently, the upfront position in structural application has been occupied by thermoset matrices, as they possess the viscosity low enough (~1 Pas) to impregnate the reinforcement without the application of considerable temperature and pressure. A number of well-established production techniques exist to manufacture thermoset composites (Figure 1). By contrast to thermosets, most thermoplastics have much higher viscosity, thus making the impregnation process rather complex. However, certain advantages of thermoplastic composites, including higher toughness, recyclability and weldability, attract the attention of both producers and customers. The high viscosity of thermoplastics can be bypassed by the use of reactive thermoplastic systems [1,2]. These systems usually consist of a monomer, activator and initiator. Due to their low molecular weight, the components of the reactive mixture exhibit almost water-like viscosity and can easily impregnate the dry reinforcement. Consequently, reactive thermoplastic systems can be used in liquid composite molding (LCM) techniques, especially in thermoplastic resin transfer molding (T-RTM). Although there is a number of reactive thermoplastic systems suitable for T-RTM (polyurethanes, polyamides (PAs), polyesters, polycarbonates, polymethylmethacrylates and others) [1], the most promising polymer for the automotive industry is PA-6 [2,3,4]. The production volumes of PA-6 are steadily growing, and reached 8 Mt in 2015. Almost 1.5 Mt of this volume was used by the automotive industry [5].

Reactive PA-6 is synthesized from a cyclic monomer called ε-caprolactam (ε-CL) via anionic ring opening polymerization (AROP) with the addition of an initiator and activator. Commercially available reactive PA-6 systems ensure a polymerization time in the range of 2–5 min. This fact together with the rapid impregnation of the dry reinforcement by the reactive mixture allows composites with the reactive PA-6 matrix to meet the demands of mass production (Figure 1). In recent years, great improvements have been achieved in the development of equipment for processing reactive thermoplastic composites [2,3,6,7]. This fact confirms an overall readiness of the industry to adopt AROP in the production of composites. Manufacturing equipment for T-RTM is produced by KraussMaffei [2,8], Engel [7,9], and some other companies. However, there are very few sources that report success in designing T-RTM molds.

The mold determines the geometry and the quality of the part, dictates the flow route of the reactive mixture, and bears the loads induced by the mold carrier and cavity pressure. It also provides the heat required to polymerize the reactive mixture. The most relevant information about the process comes from the cavity [10]. By now, considerable experience has been accumulated in the design and manufacturing of molds for various well-established LCM techniques. However, the specific nature and behavior of the reactive PA-6 system means that designing and manufacturing T-RTM molds is not a trivial case [11]. First of all, the viscosity of the reactive mixture that is injected into the T-RTM mold is well below 1 Pas for a certain amount of time. With the progression of the AROP of CL the viscosity of the reactive mixture starts to increase exponentially. However, the period of time when the viscosity is very low can be as long as 100 s [12]. To avoid the leakage of the reactive mixture during this period, the T-RTM mold must be properly sealed. Secondly, the AROP of CL occurs at high temperature (up to 200 °C) and molten CL has a highly alkaline nature [13]. Therefore, the materials of the T-RTM mold must withstand this harsh environment. Thirdly, the AROP reaction is water- and oxygen-sensitive, and therefore we must equip the mold with a special vacuum system that will assure that the medium in the mold is free of moisture and oxygen. Due to these and some other aspects of the AROP of CL, the T-RTM mold will have several unique features in its design that differentiate the T-RTM mold from all the other LCM molds.

The present study describes the process of designing a research T-RTM mold used for the production of PA-6 composite parts via the AROP of ε-CL. The results of the in-mold pressure sensor measurements are also presented and explained.

## 2. Materials and Methods

### 2.1. Materials

The reactive system for the production of samples consists of an ε-CL monomer (Brueggemann Chemicals, Heilbronn, Germany), 3 wt % sodium caprolactamate initiator (Bruggolen^®®^ C10, Brueggemann Chemicals, Heilbronn, Germany) and 3 wt % N-carbamoyllactam activator (Bruggolen^®®^ C20P, Brueggemann Chemicals, Heilbronn, Germany). The components must be dried in an oven at 30 °C for 24 h prior to experiments. Two material batches (ε-CL/initiator and ε-CL/activator) were prepared to fill the dosing units. The glass fabric used for trials has plain weave and an areal density of 310 g/m^2^.

### 2.2. T-RTM Equipment

**Figure 2:**
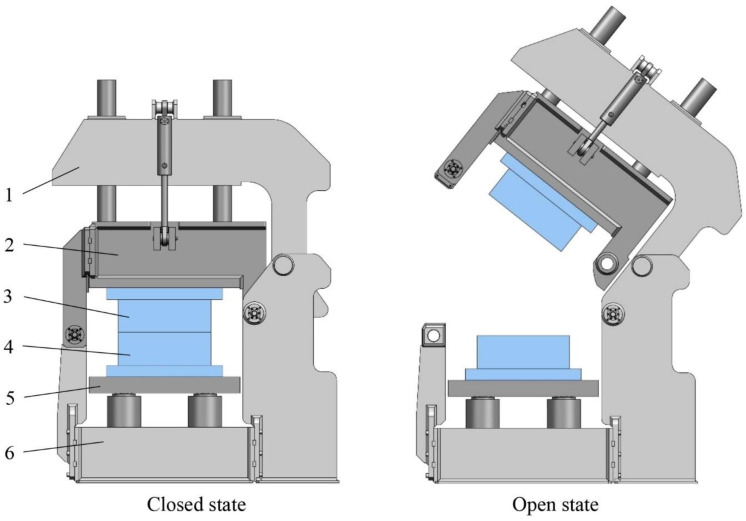
Shuttle mold carrier and a mounted T-RTM mold (KraussMaffei) (1—upper frame; 2—upper plate; 3—the upper half of the T-RTM mold; 4—the bottom half of the T-RTM mold; 5—lower plate; 6—lower frame).

A T-RTM pilot production line consists of a mold carrier, two ε-CL dosing units and a research T-RTM mold. The mold carrier and dosing units are supplied by KraussMaffei GmbH (Munich, Germany). The mold carrier (Figure 2) is a hydraulic press with a bottom stroke and a tilting upper platen. It restricts dimensions and the weight of the mold. The machine also provides a communication interface with a pressure sensor inside the mold.

The RimStar Thermo 8-8 dosing unit has a dynamic self-cleaning mixing head and is capable of increasing injection pressure to 20 MPa. This unit is used to impregnate a preform and to create the matrix. The second unit is a CometPiston machine (KraussMaffei GmbH, Munich, Germany) for processing ε-CL with various solid additives.

### 2.3. The Concept of Designing a T-RTM Mold

**Figure 3:**
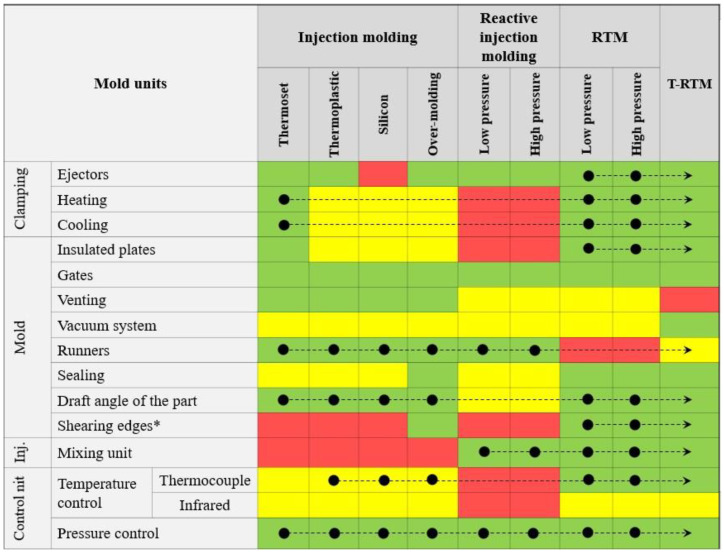
A decision-making map for T-RTM mold design (green color—a relevant unit for this type of mold; yellow color—partially relevant unit; red color—not relevant unit; black dot—the closest analogues of each unit in different molds to those of the T-RTM mold) (based on ref. [14,15,16,17]).

A T-RTM mold can barely be separated from the clamping and dosing units. The synergy of these units and the mold is even stronger than in conventional polymer processes, because the T-RTM process has certain similarities with injection molding, reaction injection molding and thermoset Resin Transfer Molding (RTM). Consequently, the design process of the T-RTM mold could contain features of the design process of the mold of the above-mentioned techniques, besides its own unique characteristics. Figure 3 demonstrates our decision-making matrix, which could serve as a road map in T-RTM mold design and development. In the decision-making matrix, we collected the information about all the standard units of closed molds (for injection molding, reactive injection molding, and thermoset RTM). We highlighted with colors the relevance of each unit in different kind of molds, and with black dots the closest analogues of each unit in different molds to those of the T-RTM mold.

The mold incorporates two mixing heads; one is dedicated to forming the part, while the second one is installed to create a class A surface on the part. The T-RTM process requires a vacuum system, a temperature and pressure controller, an oil tempering unit and a hydraulic ejector system. In the next section, we will discuss these units of the mold in more detail.

## 3. Results and Discussion

### 3.1. Details of the Design Process of the T-RTM Mold

#### 3.1.1. Product Design

**Figure 4:**
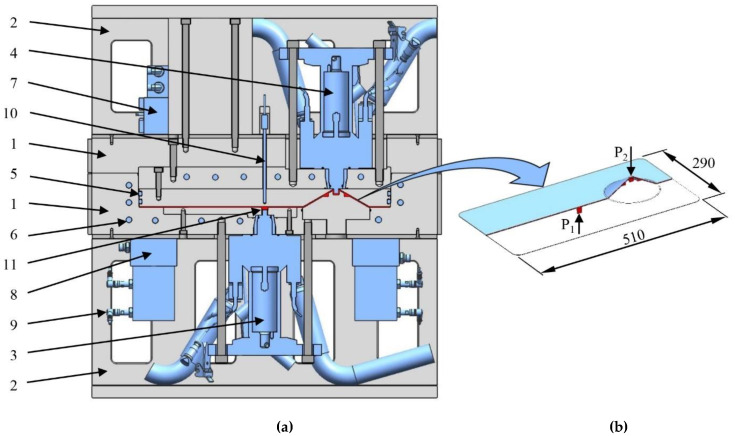
(**a**) The research T-RTM mold (1—form plates; 2—supporting structure; 3—first mixing head; 4—second mixing head; 5—sealing; 6—oil temperature controllers; 7—vacuum system; 8—hydraulic ejector system; 9—position sensor of the ejector system; 10—temperature sensor; 11—pressure sensors). (**b**) The cross-section of the formed part (P_1_—first inlet point; P_2_—second inlet point).

Any mold design process in the polymer industry starts with determining product geometry. From a research point of view, a simple plate would not satisfy our needs, because we would like to test more than just the influence of different chemical formulations, textile types, mold temperatures, pressures and dosing speeds on the output of the process. We would like to vary thickness, and create products with various architectures and surface qualities to cover typical automotive applications. We designed a total of three different demonstrators to fulfill our expectations and all these products had to be produced with the same mold. To make the explanation simpler, we divided global mold construction into two parts: the construction of the form plates (Figure 4, pos. 1), where we place the preform and produce composite; and the construction of the supporting structure (Figure 4, pos. 2), where we place auxiliary features of the mold and where we mechanically bring it into contact with the mold carrier.

#### 3.1.2. Process Requirements

As mentioned before, the mold is where we place reinforcement textiles, impregnate them and polymerize the reactive mixture. We need resin inlets to introduce the mixture into the mold. We must ensure that the reinforcement fibers will be impregnated, that the medium in the mold is free of oxygen and moisture, and that the temperature of the mold is adequate to trigger the AROP reaction. We also need to be sure that we can dose the material with an appropriate dosing rate and that the clamping force is enough to keep the mold tightly closed. As the viscosity of molten ε-CL is extremely low, we must provide appropriate sealing.

#### 3.1.3. Determining the Geometry and Structure of the Mold

After the part is designed, we calculate the overall dimensions of the mold by taking into account the most relevant processing parameters. The dimensions of the mold were limited by platen size and the geometry of the product. The range of the daylight and the opening stroke of the mold carrier limit the maximum and the minimum heights of the mold. The height of the form plates depends on product geometry and the auxiliary elements required for producing the part. There are two distinct industrial strategies for defining the dimensions of the form plates. The first approach pursues the maximum reduction of weight and leads to a decrease in robustness, while the second approach is the opposite: to create a very robust structure at the cost of sacrificing weight savings. In order to calculate the overall height of the mold, we need to know the height of the form plates and we need to meet three more requirements; the total height of the mold must be more than the minimum daylight and less than the maximum daylight plus the ejection stroke, and it should still be enough to accommodate and position the mixing heads (Figure 4, pos. 3, 4) and other units. To fulfill these last requirements, we need a supporting structure that connects the mold to the press and provides necessary space for the mixing heads and their units.

**Figure 5:**
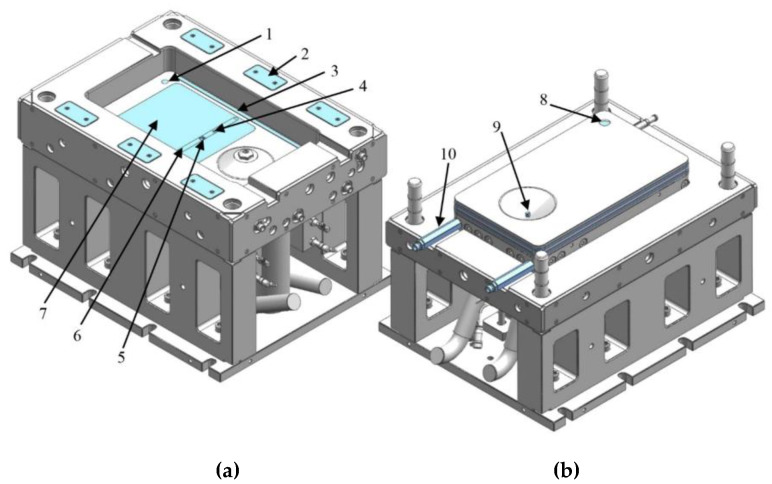
Bottom (**a**) and upper (**b**) halves of the T-RTM mold (1—ejector pin; 2—spacer; 3—textile fixer; 4—pressure sensor; 5—first injection point; 6—flow leader; 7—insert; 8—vacuum valve; 9—second injection point; 10 – oil temperature controllers).

**Figure 6:**
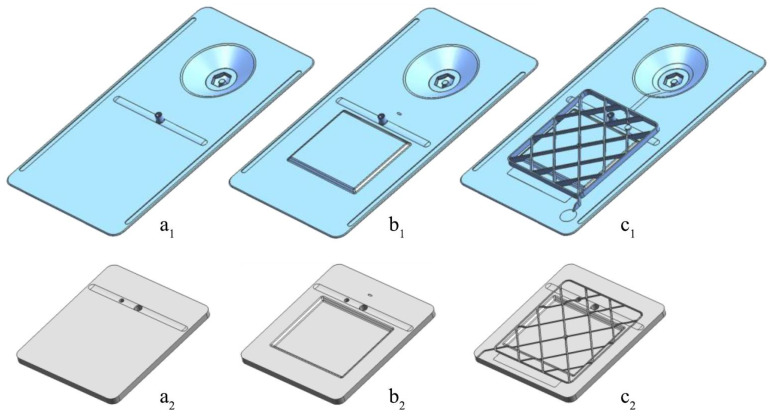
Different T-RTM parts (**a_1_**,**b_1_**,**c_1_**) and inserts designed (**a_2_**) for a flat part; (**b_2_**) for a part with a foam core; (**c_2_**) for a part with overmolded ribs.

In order to be able to vary the thickness of the product, we created a set of six spacers on the bottom part of the mold (Figure 5). The thickness range is 2–12 mm, which covers the most typical industrial applications, including the possibility of using foam cores. A set of three inserts enables the manufacturing of products with different geometries. The first insert is for the monolithic flat part (Figure 6a), the second one is for the sandwich panel (with a foam or other types of core) (Figure 6b) and the last insert is the one for producing a part with ribs (Figure 6c). Parts with ribs can be produced either in one shot or in two shots (overmolding).

We also used an innovative approach in the sealing of two mold halves. Traditionally, in RTM and reaction injection molding molds, the sealing contour is parallel to the product, which limits the ability to vary thickness. In our mold, we used a double seal (Figure 4, pos. 5) in the plane perpendicular to the cavity, creating a kind of a core–cavity connection. Both seals slide inside the bottom half of the mold, making it possible to vary part thickness.

To establish a fully homogeneous temperature in the mold, we designed a conventional cooling circuit for oil heaters (Figure 4, pos. 6).

**Figure 7:**
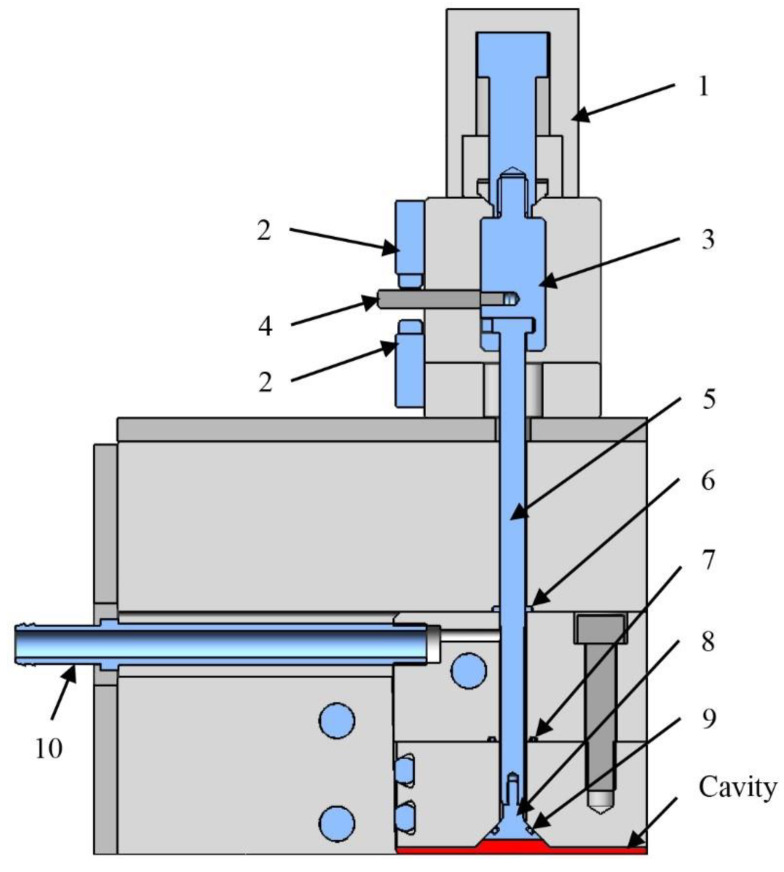
The vacuum system of the T-RTM mold (1—hydraulic cylinder; 2—positioning sensor; 3—coupling; 4—rod; 5—valve shaft; 6, 7, 9—sealing; 8—valve head; 10—vacuum connector).

The AROP reaction is water- and oxygen-sensitive, therefore we had to assure that the medium in the mold would be free of moisture and oxygen. For that, we decided to apply a vacuum and equipped the mold with a special vacuum system (Figure 7). Our vacuum system in the mold differs from those we can use in standard injection molds. Its distinct feature is a cone-shaped valve head (Figure 7, pos. 8), which opens and closes the vent channel with a hydraulically operated mechanism. It makes it possible to create a vacuum in the cavity and to shut it off before the flow reaches the vacuum outlet. The valve position is indicated by the two position sensors (Figure 7, pos. 2). The valve head (Figure 7, pos. 8) is also sealed. We selected the hydraulic system to operate a tightly sealed valve because it creates sufficient opening and closing forces.

**Figure 8:**
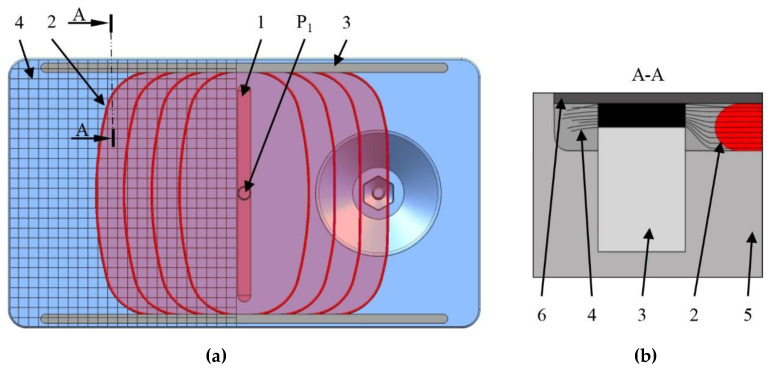
Layout of the mold cavity with the reinforcement inside (**a**) (P_1_—first inlet point; 1—flow leader groove; 2—flow front distribution in the cavity; 3—textile fixer; 4—textile; (**b**) cross-section of the textile fixer (5—the upper half of the T-RTM mold; 6—the bottom half of the T-RTM mold).

We can create an additional coating on the surface of the part by means of opening the mold a little bit and creating a space for the second shot. We therefore created two independent inlet points in the mold. The first inlet point (Figure 4, P_1_) is located in the bottom half of the mold and connected to the mixing head (Figure 4, pos. 3) that doses the material for forming the part. It is placed in the flow leader groove (Figure 8, pos. 1), which helps make the flow front more uniform in the flow direction. The flow leader can be filled quickly and from there the melt starts to distribute crosswise in both directions. The second inlet point (Figure 4, P_2_) is in the upper mold half and connected with the mixing head (Figure 4, pos. 4) that creates the coating. The second inlet point is located in a cone that has a counterpart in the stationary mold half. The height of the counterpart cone can be adjusted (Figure 5). The latter feature is necessary to maintain a constant space between the mold halves after the thickness is changed. To form a coating a few tenths of a millimeter thick, we need a gap of 1–2 mm and the second shot, as well as additional external pressure, which forms the upper layer.

In thermoset RTM, there is a well-known issue caused by imperfections on textile preform edges. Some textiles tend to fray, which leads to a lower density at the edges and makes it easier for the melt or resin to go first “around” the preform. This phenomenon is called “race tracking” and it results in dry spots in a product. To avoid such defects, we placed slightly bigger plies, and are able to hold the preform edges with special silicone textile fixers (Figure 8, pos. 3). This helps to avoid the race tracking effect by guiding the flow to the center of the cavity. The fixers create a higher resistance for the melt flow, forcing it to go first through the preform and impregnate the edges only at the end. We placed these fixers perpendicular to the flow leader.

To measure cavity pressure, we selected a piezoelectric sensor (6161AA 2, Kistler Instrumente GmbH, Sindelfingen, Germany). This type is used in thermoset RTM. The pressure sensor (Figure 4, pos. 11) is located as close as possible to the first inlet point. This location allows feedback almost immediately after dosing starts, and allows to observe the pressure drop during the entire filling and polymerization stage. The temperature of the mold is controlled with the help of a conventional PT-100-type thermocouple (Figure 4, pos. 10), embedded in the upper mold half close to the surface of the cavity.

**Figure 9:**
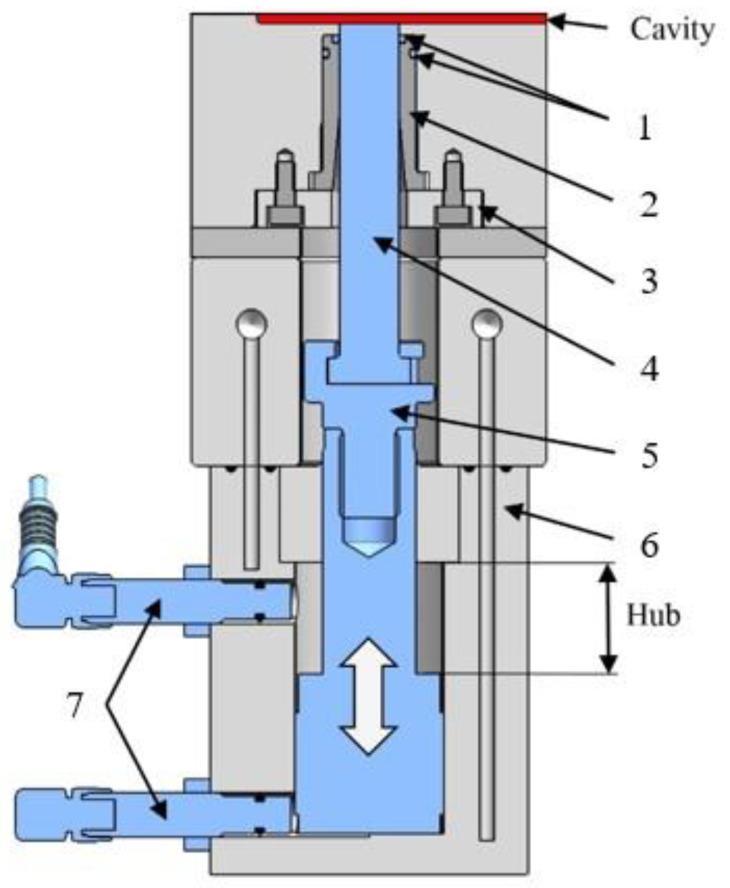
Ejector system (1—sealing; 2—guide bush; 3—fixing plate; 4—ejector pin; 5—coupling; 6—hydraulic cylinder; 7—position sensor).

The last step in T-RTM is the demolding of the product. To do it in an automated way, we need an ejector system (Figure 4, pos. 8; Figure 9). Normally, ejectors are embedded between two plates operated by the machine. However, the built-in mixing heads with their tubes limit the available free space for the ejector unit. Therefore, in our case, each ejector is operated by a separated hydraulic cylinder and each cylinder is fed by the same oil circuit to ensure their parallel movement.

#### 3.1.4. Mold Materials Selection

We selected stainless steel as the main material for the form plates, as it can withstand the corrosive environment under the high temperature and pressure loads in T-RTM molds. Inserts for different architectures are made from aluminum. Thermal insulation of the mold is provided by the insulation plates made from BRAGLA^®®^ N (Brandenburger Isoliertechnik GmbH. & Co., Landau in der Pfalz, Germany). This insulation material has a low thermal conductivity (0.22 W/(m∙K)), while its operation temperature can be as high as 350 °C. Moreover, all the seals in the T-RTM mold must also withstand a maximum temperature of 200 °C, a maximum pressure of 20 MPa and an aggressive alkaline medium. We selected the Viton^TM^ fluoroelastomer (DuPont, Wilmington, DE, USA) to seal the T-RTM mold, because this material fulfills all the above-mentioned requirements.

### 3.2. Testing of the T-RTM Mold

We produced both neat and fiber-reinforced samples with reactive PA-6 at various dosing speeds (20, 30 and 40 g/s). The temperature of the mold was kept at 150 °C during the tests. The dosing volume was 280 g for reinforced samples and 365 g for the unreinforced samples. We used half of the mold volume as a buffer to keep the nitrogen bubbles outside of the reinforced zone (Figure 8). We produced three samples for each material combination and with each dosing rate to check the stability of the process.

**Figure 10:**
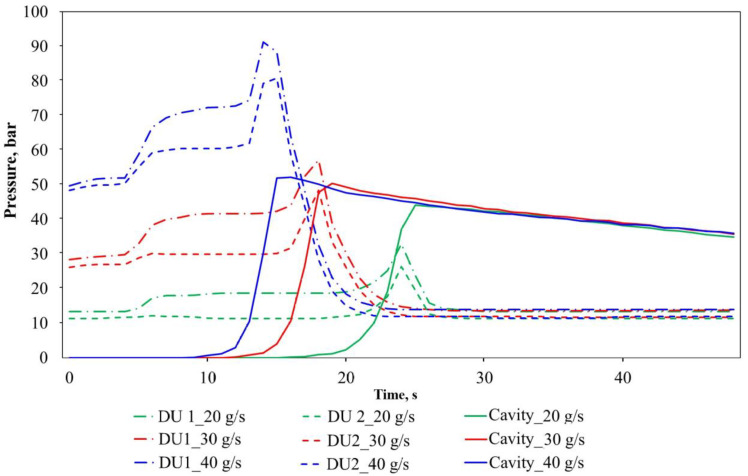
The pressure curves of the neat PA-6 during the T-RTM process (DU1 and DU2 are dosing unit 1 and dosing unit 2, respectively).

The in-mold pressure measurement results for neat PA-6 are presented in Figure 10. After reaching the peak value, the in-mold pressure starts to decline because the volume of the reactive mixture decreases in the mold, which indicates polymerization and crystallization in the mold. Moreover, with all three dosing rates, these parts of the curves are very close to each other and seem to be independent of dosing speed. This fact shows that the rate of polymerization is not affected significantly by the dosing rate. Further investigations at various mold temperatures can extend this knowledge.

**Figure 11:**
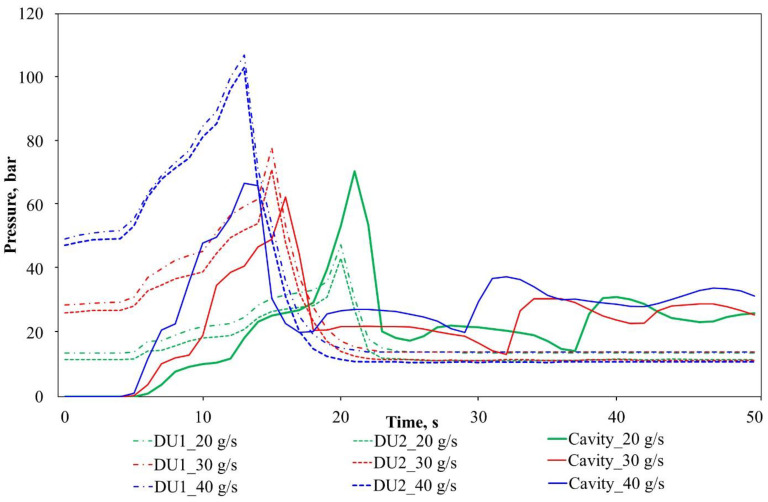
The pressure curves of the reinforced PA-6 during the T-RTM process.

Polymerization and crystallization behavior might be affected by the fabric placed in the mold, as the in-mold pressure curves changed after reaching the maximum pressure (Figure 11).

We observed significant differences between the in-mold pressure curves of reinforced and neat samples. To explain this phenomenon, further investigation of the T-RTM process at various temperatures is required. With the current mold configuration, we can only identify the temperature and pressure at one point in the cavity. As such, in-mold process control performed through in-mold pressure feedback would be preferable, as many physical and chemical processes take place in the mold simultaneously. Furthermore, the number of sensors should be increased so that more important data can be collected, and the process can be controlled and monitored more precisely.

## 4. Conclusions

In this study we proposed a design concept and developed a prototype mold for the T-RTM process. The mold has been designed to manufacture parts with various features and thicknesses. The range of these thicknesses can vary between 2 and 12 mm, which covers the most typical automotive applications. The mold developed enables the production of neat polymer parts, fiber-reinforced composites, and sandwich structures with various cores. We can add ribs to the part, and these ribs can be formed either together with the part in one shot or by overmolding on the base part in a second shot. Additionally, we can apply in-mold coating through a second inlet, by which we can form an extra layer of material via compression molding.

A number of engineering problems that appear in the T-RTM process were solved. We proposed special designs for the vacuum system and the ejector unit of the mold. We also developed an appropriate sealing to prevent leakage of the low-viscosity mixture, and introduced pressure and temperature sensors to control the whole process.

We also indicated that the preform placed into the mold has a significant effect on the T-RTM process. The preform changed the pressure profile during the cycle and the reason behind this is either the influence of the fabric on the AROP reaction, or the influence of the fabric on the measurement of pressure itself. To separate these effects, further investigations are required.

To sum up, in the first round of development, we aimed to introduce a concept of T-RTM mold design as well as demonstrate the operability of the developed mold. The mold can be used for further investigation and a deeper analysis of the T-RTM process, and that is the next step of our research.

## Abbreviations

AROP	anionic ring opening polymerization
HP	high pressure
LCM	liquid composite molding
LP	low pressure
PA	polyamide
RIM	reactive injection molding
T-RTM	thermoplastic resin transfer molding
ε-CL	ε-caprolactam
